# Modern pharmacological treatment of obese patients

**DOI:** 10.1177/2042018819897527

**Published:** 2020-01-22

**Authors:** Marcus May, Christoph Schindler, Stefan Engeli

**Affiliations:** Hannover Medical School, MHH CRC Core Facility, Feodor-Lynen-Strasse 15, Hannover, 30625, Germany; MHH Clinical Research Center Core Facility (OE 8660) and Center for Pharmacology and Toxicology, Hannover, Germany; Hannover Medical School, Institute of Clinical Pharmacology, Hannover, Germany

**Keywords:** drug treatment, obesity, pharmacokinetics, weight gain, weight reduction, weight reduction surgery

## Abstract

There are many angles to consider in drug treatment of obese patients. On the one hand, some specific weight loss drugs are available, on the other, several drugs are associated with unintentional weight changes. When treating an obese patient for any given disease, several physiological changes may influence the pharmacokinetic properties of the drugs required. Thus, increased body weight may influence the efficacy and safety of some drug treatments. Even more complicated is the situation after weight reduction surgery. Due to the various changes to the gastrointestinal tract induced by the different surgical techniques used, and the dynamic changes in body composition thereafter, drug dosing has to be constantly reconsidered. Whereas all of these issues are of clinical importance, none of them have been investigated in the necessary depth and broadness to ensure safe and efficacious drug treatment of the massively obese patient. Individual considerations have to be based on comorbidities, concomitant medication, and on specific drug properties, for example, lipophilicity, volume of distribution, and metabolism. In this article we summarize the data available on different aspects of drug treatment in the obese patient with the hope of improving patient care.

## Introduction

More than 1.9 billion adults worldwide are overweight, including over 650 million with clinically relevant obesity, and the number of obese patients continues to rise.^1^ Most likely, this development will not change substantially in the foreseeable future due to the absence or failure of preventive measures. Obesity is associated with increased mortality and comorbidity, due, in part, to specific obesity-associated diseases such as type 2 diabetes mellitus, hypertension, cardiovascular diseases, respiratory dysfunction, some carcinomas, nonalcoholic fatty liver disease, and orthopedic degenerative diseases.^2,3^ But clinical management of obese patients is often complicated whether the disease is obesity-associated or not. Specific guidelines for weight-adjusted dose modifications are lacking for almost all drugs available, although there is considerable concern that some drugs, especially those with a narrow therapeutic range, might require dose adjustment in obese patients. There is also only scant evidence on the effectiveness of drugs used regularly in obese patients. One reason for the lack of data is underrepresentation of patients with obesity in clinical trials.^4,5^ In summary, physicians should be aware of how current pharmacotherapy influences metabolism and body weight, and how pharmacotherapy is influenced by obesity.

**Figure 1.**
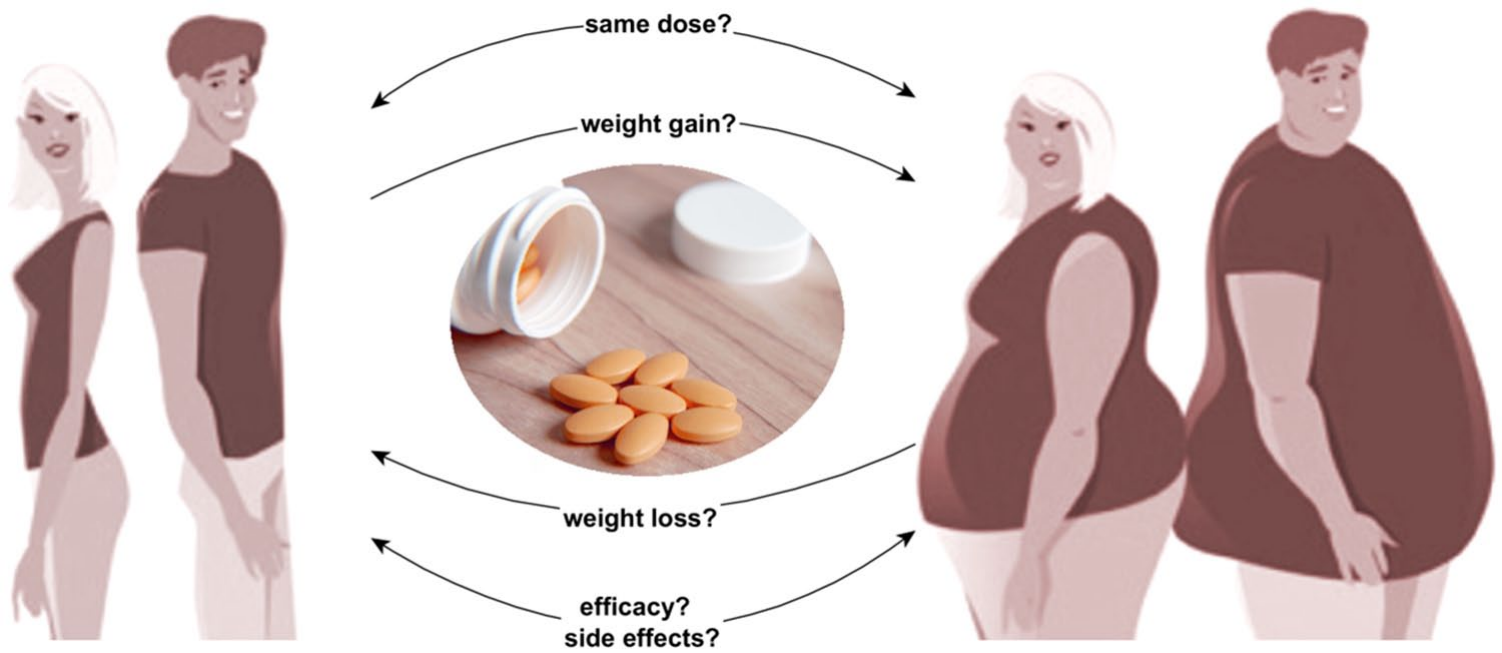
Linkage of drug treatment and body weight. Questions to be addressed when pharmacotherapy is planned in obese patients: Can the drug be given in the same dose as in normal weight patients? Does the drug promote weight gain or weight loss (might there be an alternative drug with better metabolic properties in obese patients). Are efficacy and side effects of the drug independent of body composition?

The relationship between body weight and drug therapy is characterized by the following four main topics,^6^ which are also illustrated in Figure 1:

1. Drugs intentionally used to reduce body weight
2. Drugs causing unintentionally weight changes as a side effect
3. Pharmacokinetic alterations due to increased body weight
4. Influence of body weight on drug treatment efficacy

In recent years, several weight-reducing drugs received marketing authorization, and there are several current reviews available summarizing this topic.^7–11^ However, data concerning weight changes as side effects of drugs, pharmacokinetic alterations in obese patients, and influence of body weight on drug efficacy are rather rare.

Here, we summarize relevant information to allow evidenced-based individual pharmacotherapy of obese patients. Clinically relevant drug characteristics of available anti-obesity drugs, important drugs promoting the development of obesity, and changes in pharmacokinetics and pharmacodynamics in relation to body weight are presented.

## Antiobesity drugs

Several drugs have been developed specifically to reduce body weight; some of these have gained marketing authorization during the last 5 years, whereas several others are still in development.^8^ Besides that, weight loss is an important side effect of some commonly used drugs. Well-known examples include Topiramate, an antiepileptic drug that is also used for migraine prophylaxis, Roflumilast in chronic obstructive pulmonary disease (COPD) therapy, and Bupropion and Fluoxetine in the treatment of depression. For some other drugs, the weight-reducing effect is not the primary treatment indication, but is desirable, and probably explains part of the effectiveness. Important examples are sodium-glucose transport protein 2 (SGLT2) inhibitors and glucagon-like peptide-1 receptor (GLP1) agonists when used to treat type 2 diabetes mellitus.^12,13^ In some diseases where several drugs are available, a specific drug may be chosen because of its known weight-lowering side effect, for example, Topiramate seems to be a good treatment option for obese patients with migraine. But weight-reducing effects may also lead to off-label use of some drugs, and abusive use of some of these drugs cannot be ruled out, for example, Methylphenidate.^14^

Of course, not every obese patient needs pharmacological treatment for weight loss. Exercise, diet, and behavior modification should always be the cornerstones of anti-obesity treatment.^15,16^ However, many affected patients do not lose weight, or fail to maintain weight loss, with that approach. There is consensus that anti-obesity drug treatment may be considered for individuals who fail to respond to lifestyle interventions after 6 months of treatment, and have a body mass index (BMI) of >30 kg/m^2^ or a BMI of >27 kg/m^2^ with weight-associated comorbidities.^15,17,18^ However, weight reduction *per se* should not be the main goal of treatment. Improvement of obesity-associated comorbidities like hyperglycemia, hyperlipidemia, and hypertension are at least of equal importance. However, expectations in regard to weight loss efficacy are often very unrealistic. Patients and healthcare providers should realize that efficacy of available anti-obesity drugs is often limited to a reduction of 5–10% of body weight over a 1-year period. Drug-induced weight loss typically does not occur for more than 6–8 months. Obesity is a chronic disease and requires long-term treatment. Many patients and health care providers still do not act according to this concept. No one would suggest discontinuing antidiabetic medication when hemoglobin A1c (HbA1c) is improved after a new medication was started. Regarding obesity, there is regular dispute about regained weight after anti-obesity medication was discontinued, which demonstrates the need for effective weight maintenance strategies. As with other chronic diseases, anti-obesity medication should be viewed as a next-step treatment option on basis of a continuous healthy lifestyle regime including increased daily activity and a calorie-deficit diet. Drug therapy should never be a standalone therapy, or even universal remedy, against obesity. Pharmacotherapy can be considered as an adjunct to bariatric surgery to maintain weight and prevent weight regain some time after surgery. In some cases, added drug treatment can even facilitate further weight loss in these patients.^19^

### Anti-obesity drugs in Europe

There is a wide range of Anti-obesity drugs in Europe, from amphetamine-type medicines to preparations made from algae and homeopathic medicines. However, only a few of these drugs are recommended in current guidelines.^15,16^ Anti-obesity drugs have been used for more than 100 years.^7^ Some of the drugs still available activate the sympathetic nervous system similar to the action of amphetamines. These amphetamine-type drugs can cause cardiovascular and psychological adverse effects. Moreover, the product information summary contains warnings regarding pulmonary arterial hypertension and addiction potential when taken over a long period of time. These drugs may be effective in some patients, but are authorized only for short-term use (4–12 weeks), which does not fit into a well-structured and long-term effective obesity therapy. Moreover, type 2 diabetes mellitus is a contraindication for sympathomimetic drugs, and, therefore, many patients who seek weight reduction therapy are not suitable for this kind of treatment. Another factor is the lack of safety data from large randomized controlled trials. One exception may be Cathin (=Norpseudoephedrine), because at least a small randomized controlled trial was conducted recently.^20^ Some of the available anti-obesity preparations contain relevant amounts of ethanol or iodine, which can be a risk in patients with alcohol dependency or thyroid gland diseases. Swelling agents from algae or crustacean-derived chitin products to bind dietary lipids may reduce absorption of other drugs like oral contraceptives or thyroid hormones. Thus, prescription or recommendation of these drugs and preparations requires a considerable knowledge about their properties and constituents.

Of the drugs with marketing authorization for the treatment of obesity in Europe, sufficient efficacy and safety information from large randomized controlled trials are available for only Orlistat, Liraglutide, Bupropion/Naltrexone, and, with some limitations, Cathin.

### Orlistat

With Orlistat, an average weight reduction of about 3.8 kg above placebo was seen in clinical trials. In patients with type 2 diabetes mellitus, weight reduction was about 2.5 kg.^21^ The observed weight reduction was associated with a reduction in blood pressure; however, cardiovascular endpoint studies with orlistat are still lacking.^22^ The incidence of type 2 diabetes mellitus is lowered in obese patients with impaired glucose tolerance when treated with orlistat compared with lifestyle interventions alone.^23^ Orlistat is a lipase inhibitor, reducing dietary fat uptake in the small intestine by about 30%. Thus, efficacy and side effects depend on daily fat intake. Intestinal side effects (fatty/oily stool, fecal urgency, oily spotting, increased defecation, fecal incontinence, flatus with discharge, and oily evacuation) are common problems with Orlistat. Concomitant use of natural fibers (psyllium mucilloid) might help to ameliorate these adverse gastrointestinal effects.^24^ Orlistat does not affect cytochrome P450 metabolism, but drug interactions are possible due to resorption inhibition in the small intestine. For example, this interaction is well documented for concomitant medication with ciclosporin.^25^ Resorption of fat-soluble vitamins might also be reduced, and oral multivitamin supplementation should be considered during treatment.^26,27^ Currently, Orlistat is the only marketed anti-obesity drug of this category (lipase inhibitor) in Europe. Cetilistat (ATL-962) is equipotent to Orlistat regarding fecal fat excretion, but seems to have a somewhat better gastrointestinal side effect profile. Cetilistat has gained marketing authorization in Japan, and an approval in the US is expected.^8^

### Liraglutide

Liraglutide is a long-acting glucagon-like peptide-1 (GLP1) receptor agonist to be injected subcutaneously once daily. Marketing authorization was granted by the European Medicines Agency (EMA) in 2009 in doses up to 1.8 mg for the treatment of type 2 diabetes mellitus under the tradename Victoza®. In 2015, marketing authorization was granted for treatment of obesity in doses up to 3 mg per day, the corresponding tradename is Saxenda®.^7,9^ The SCALE Obesity and Prediabetes trial evaluated Liraglutide in obese and overweight nondiabetic patients. The study included 3731 subjects who were treated with Liraglutide 3 mg daily or placebo. Patients were also counseled on diet and exercise. At the end of the 56-week trial, the Liraglutide group lost an average of 8% (7.2 kg) of their initial body weight compared with 2.6% (2.8 kg) in the placebo group. The placebo-corrected weight loss was 4.4 kg.^28^ The proportion of patients achieving a weight reduction of 5–10% of baseline weight is 2–3 times greater with Liraglutide than with placebo.^28,29^ For weight loss, there is a clear dose relationship for doses up to 3 mg daily.^30^ The recommended dosing scheme is to start with 0.6 mg daily, and then increase the dose by 0.6 mg weekly up to the target or maximum dose, to reduce the frequent gastrointestinal side effects.^8,9^ Cardiovascular safety is of utmost importance for new anti-obesity drugs, especially after the withdrawal of Sibutramine due to the increased risk of cardiovascular adverse effects.^31^ Treatment with Liraglutide lowers waist circumference, lipids, HbA1c, blood pressure, blood sugar, and insulin to an extent proportional to the achieved weight loss. Moreover, the LEADER study demonstrated, for the first time for treatment with a GLP1 receptor agonist, a reduction in cardiovascular and total mortality in patients with type 2 diabetes mellitus.^32^ Although weight reduction was not a primary endpoint in the LEADER trial, treatment with Liraglutide resulted in a weight loss of 2.3 kg over placebo. Still, long-term efficacy and safety of GLP1 receptor agonists remain somewhat controversial. Even if cardiovascular protection seems to be a class effect, the suspected risk of worsening of diabetic retinopathy is still in need of clarification.^32^ The risk of pancreatitis and cholecystitis is now largely considered refuted, but long-term data are lacking for Liraglutide and other GLP1 receptor agonists for the treatment of obesity. Animal studies with Liraglutide raised the suspicion of an association with medullary thyroid cancer. Even though there has been no sign of this association in humans, a personal or family history of medullary thyroid cancer or multiple endocrine neoplasia type 2 is considered a contraindication for treatment with this drug.^33^ Liraglutide is a useful therapeutic option to achieve weight loss in individual subjects, especially in patients with type 2 diabetes mellitus. However, therapy costs may be too high for many patients, which might limit the broader use of these drugs depending on the reimbursement situation in a specific country.

There are several more long-acting GLP-1 receptor agonists either already licensed for treatment of type 2 diabetes mellitus or currently in clinical trials. Semaglutide is currently being investigated as weight loss therapy and will be available in the future in an oral application form as well.^34,35^

### Bupropion/naltrexon

The fixed combination of naltrexone and sustained release bupropion was approved by the United States Food and Drug Administration (FDA) in 2014 under the tradename Contrave®, and by the EMA in 2015 as Mysimba®.^8^ The combination drug naltrexone/bupropion seems to have a lower weight-reducing efficacy than the previously described Liraglutide, which is countervailed by the combined spectrum of adverse effects of both drugs.^36^ Bupropion reduces appetite and increases energy expenditure through increased dopamine activity and proopiomelanocortin (POMC) neuronal activation, modest dose-dependent weight lowering efficacy has been shown in several clinical studies.^37,38^ The overall safety profile of Bupropion is good, with only a mild rise in blood pressure and mild tachycardia reported in some patients, and a substantially reduced risk of seizures with the sustained release Bupropion.^36^

Long-term efficacy of bupropion is inhibited by a negative feedback on pro-opiomelanocortin (POMC) neurons *via* opioid receptors, which, in turn, is blocked by Naltrexone. Naltrexone failed to produce consistent or clinically meaningful weight loss, but combined bupropion/naltrexone induced significantly greater weight loss over 56 weeks compared with monotherapy and placebo.^36,39^ Although the fixed-dose combination may be helpful in helping some obese patients to reduce weight, the lack of long-term and cardiovascular safety data limits the use of this drug in patients with increased cardiovascular risk.

### Cathin

Cathin is one of the remaining agents from the era of amphetamines and amphetamine-type drugs that were used in the past 40 years for the treatment of obesity.^20^ Cathin is an alkaloid isolated from khat leaves, and is licensed for the short-term treatment of diet-related obesity. Pharmacologically, Cathin acts as an amphetamine-type agent and induces anorexia, increased alertness, increased sensory stimulation, hyperthermia, increased respiration and heart rate, rise of blood pressure, constipation, and urine retention.^20^ In a recent randomized controlled trial with 241 overweight and obese patients, treatment with three doses of Cathin (16 mg, 32 mg, 53.3 mg) was compared with placebo over a treatment period of 24 weeks. Patients treated with Cathin had significantly greater weight loss compared with placebo over 24 weeks (6.5 ± 4.2 kg; 6.2 ± 4.7 kg; and 9.1 ± 5.4 kg respectively *versus* 2.4 ± 4.4 kg for placebo). A modest dose-dependent increase in heart rate was observed in the treatment groups, but otherwise no safety problems were detected. Overall dropout rate was highest in the placebo group. A therapy over 24 weeks with Cathin seems to be safe, with a considerable efficacy, but the long-term benefit-risk ratio still remains unclear, and further exploration in larger clinical trials is needed, especially with respect to cardiovascular safety.^20^

### Antiobesity drugs approved by the FDA

In addition to the above-mentioned anti-obesity drugs available in Europe, the selective serotonin receptor agonist Lorcaserin (tradename Belviq®) and the combination drug Phentermine/Topiramate (tradename Qsymia®) are approved by the FDA for treatment of obesity in the United States.^7–9^

Lorcaserin reduces appetite by binding to the 5-HT2C receptors, for which it has a ~15–100 fold greater selectivity over 5-HT2A and 5-HT2B receptors. Efficacy and safety was shown in the BLOSSOM and BLOOM trials. Patients treated with Lorcaserin lost significantly more body weight compared with placebo (−5.8% *versus* −2.5%). Headache, upper respiratory tract infections, and nasopharyngitis were common reported side effects. Importantly, cardiac valvulopathy rates were similar to placebo. Significant improvements in lipid and glycemic indicators, quality-of-life measures, and vital signs were seen in the Lorcaserin group compared with placebo.^40^

Phentermine belongs to the class of amphetamine-type drugs and promotes weight loss by activating the sympathetic nervous system, thus increasing resting energy expenditure and decreasing food intake. Phentermine is contraindicated in patients with cardiac comorbidities, hyperthyroidism, and glaucoma, and in patients who have taken monoamine oxidase inhibitors within 14 days. The second drug in Qsymia® is topiramate, which is approved for the treatment of epilepsy and migraine prophylaxis. The observed weight-reducing side effects are presumably triggered by induced taste aversion, and, thus, decreased caloric intake. Topiramate is teratogenic and thus contraindicated during pregnancy; women of child-bearing potential must take contraceptive measures.^41–43^ Efficacy and safety of the phentermine/topiramate combination was shown in the EQUIP, Conquer, and Sequel trials. A dose-dependent placebo-subtracted weight loss of up to roughly 9% was reported after 1 year of treatment. Adverse side effects included paresthesia, dizziness, dry mouth, constipation, dysguesia, insomnia, and anxiety. Improvements in lipid parameters and blood pressure, as well as improvement of fasting glucose, insulin levels, and reduced progression to type 2 diabetes mellitus were seen in the clinical studies.^41–43^ A specific cardiovascular safety trial has not been conducted to date.

### Perspective

Semaglutide and exenatide are both GLP-1 receptor agonists with marketing authorization for the treatment of type 2 diabetes mellitus. Both drugs are in development for the treatment of obesity.^8^ While exenatide is a short acting GLP-1 analog, and, thus, does not provide substantial treatment benefits in comparison with liraglutide, semaglutide is a long acting GLP-1 analog with a once per week subcutaneous treatment (0.25 mg up to 1.0 mg). Moreover, an oral application is also in development, which would make the drug much more attractive for patients.^34^ Both semaglutide and exenatide provide comparable or even more efficacy than does treatment with liraglutide. Both drugs are in phase II trials, and marketing approval for obesity can be expected in the near future.^8^ There are several other agents under investigation, targeting special pathways involved in the development of obesity. Examples of promising agents are: melanocortin-4 receptor agonists (RM-493), NPY-inhibitors (the Y2/Y4 receptor agonist obinepitide and a selective Y4 receptor agonist TM30339), sympathomimetics (tesofensine), combinations of GLP-1 and PYY3-36 agonists, lipase inhibitors with lesser side effects (cetilistat, marketing authorization in Japan already granted), β3-adrenoreceptor agonists (LY-377604 + Sibutramin), angiogenesis inhibitors (ALS-L1023), sirtuin 1 (SIRT1) activators, molecules targeting cGMP pathways (sildenafil and linaclotide), and several dual receptor agonists like tirzepatide, which is a promising dual GIP (Gastrin Inhibitory Peptide) and GLP-1 receptor agonist currently in development.^8,44–46^ These drugs present only a few candidates in the obesity drug pipeline.^45^

## Common drug classes associated with weight changes

In daily clinical practice, patients may complain about treatment-associated weight gain. The number one weight-increasing medicine class on the list are atypical antipsychotics, along with systemically applied glucocorticoids and insulin or sulfonylureas for lowering of blood glucose.^47–49^ Concrete knowledge about this topic often remains anecdotal, because changes in body weight are often ignored during drug registration studies. On the one hand, every medicine with a sedative effect may promote weight gain due to possibly reduced daily activity; on the other, drugs with central nervous system (CNS) depressant effect often display a broad influence on a variety of neurotransmitter receptors and transporters. A pathophysiologic connection can be assumed only in cases where drug influences on hunger, satiety and energy metabolism are known. Unfortunately, for most drugs, such data are not available. Thus, only observations in clinical studies or registries can be taken as evidence for weight effects. Current knowledge about weight gain with selected drugs as reported in clinical trials is summarized in Table 1. In the table, we also name drugs within the same indication with more favorable weight effects, but this is not meant as an explicit treatment recommendation. Weight effects of certain drugs should be kept in mind, especially when obese patients are treated. However, of course this cannot be the only selection criterion. Whether a weight-neutral or even weight-reducing drug is suitable as a therapeutic alternative should be decided from the clinical point of view for the individual patient.

**Table 1.** Reported drug influence on body weight in clinical trials.

	Drugs leading to weight gain	Weight gain in kg	Possible alternative	Weight loss in kg
Antiepileptics/mood stabilizer	Valproate	1.2–5.8^a^	Zonisamide	−7.7
	Gabapentin	2.2	Topiramate	−3.8
	Lithium	4.0^a^	Lamotrigine	±0
	Carbamazepine	1.0^a^		
Neuroleptics	Olanzapine	2.4	Ziprasidone	−3.2 to −2.7^a^
	Quetiapine	1.1		
	Risperidone	0.8		
	Clozapine	4.2 to 9.9^a^		
	Aripiprazole	0.6^a^		
Glucocorticoids	Class effect with approximately 4–8% increase in body weight			
Antidiabetics	Insulin	1.8–6.5^a^		
	Glimepiride	2.1	Metformin	−1.1
	Glibenclamide	2.6	Acarbose	−0.4
	Pioglitazone	2.6	GLP1-agonists	−1.2 to −5.6
	Tolbutamide	2.8	SGLT2-Inhibitors	−2.2 to −4.7
	Sitagliptin	0.55		
	Nateglinide	0.3		
Antidepressives	Nortriptyline	3.7^a^	Bupropion	−1.3
	Doxepine	2.7^a^	Fluoxetine	−1.3
	Amitriptyline	1.8	Sertraline	(unknown)
	Mirtazapine	1.5	Venlafaxine	(unknown)
			Duloxetine	(unknown)
Betablockers	Atenolol	1^a^	(ACE-Inhibitors)^b^	+/–0
	Metoprolol	0.5–1.5^a^	(AT1-Blockers)^b^	+/–0
	Propranolol	−0.6 to 2.3^a^	(Thiazides)^b^	+/–0

Adapted from Pilitsi and colleagues,^8^ Domecq and colleagues,^37^ and Leslie and colleagues.^48^

a Limited or no data available from randomized placebo controlled trials and measured weight change.

b No effect on body weight but with metabolically favorable or at least neutral (thiazides) profile.

ACE, angiotensin-converting enzyme; AT1-Blockers, angiotensin II receptor antagonists; GLP1, glucagon-like peptide-1 receptor; SGLT2, sodium-glucose transport protein 2.

### Antidiabetics

Influence of drugs on body weight plays a major role, particularly in the treatment of type 2 diabetes mellitus, since weight gain is strongly associated with disease progression and many antidiabetic drugs promote weight gain.^50^ Insulin, and insulin analogues, stimulate weight gain more than oral antidiabetics. In clinical trials, mean weight gain on insulin therapy has been between 1.8 and 6.5 kg.^48^ Various mechanisms seem to play a role here, such as increased rate of hypoglycemia, decreased energy loss through the kidneys by blood glucose normalization, and the anabolic effect on muscle and adipose tissue. Also, central insulin effects as well as the unphysiological application into subcutaneous adipose tissue are discussed as possible mechanisms.^50^ Presumably due to stimulation of endogenous insulin secretion, sulfonylureas also cause a considerable amount of weight gain. Thiazolidinediones, such as Pioglitazone, have also been shown to increase body weight. In contrast, alpha-glucosidase inhibitors, as well as dipeptidyl-peptidase-4 inhibitors, are weight-neutral, and metformin, SGLT-2 inhibitors, and glucagon-like peptide-1 agonists decrease body weight in ascending efficacy.^48,50,51^

### Antipsychotics

Antipsychotics, with their variety of neurotransmitter-receptor affinities, have varying effects on body weight.^48^ In general, medications in the class of typical antipsychotics usually show a greater weight gain, which is most pronounced when patients are treated with Thioridazine. Among the drug class of atypical antipsychotics, especially clozapine, olanzapine, and risperidone are associated with increased body weight during therapy. In this drug class, only Ziprasidone is not associated with the development of obesity. Lithium, which is used as a mood-stabilizing agent in bipolar disorder, also often leads to an increase in body weight.^48^

### Antidepressants

Tricyclic antidepressants are associated with weight gain. This is especially true for Amitriptyline, Clomipramine, Doxepin, and Imipramine. Likewise, monamine oxidase inhibitors lead to an increase in body weight. Newer antidepressants such as selective serotonin reuptake inhibitors, selective norepinephrine reuptake inhibitors, and Mirtazapine are less likely to increase body weight, and sometimes patients even lose weight during therapy.^52^

### Antiepileptics

Valproate and Carbamazepine are associated with weight gain, likewise Gabapentin and Pregabalin. Only in patients treated with Topiramate and Zonisamide was this undesirable drug effect not observed.^53^

### Immunosuppressants

Of the drugs used for immunosuppressive therapy, glucocorticoids especially, but also specific calcineurin inhibitors such as Tacrolimus and mTOR inhibitors like Sirolimus, increase body weight.^48^

### Other drugs

Contrary to the popular belief that therapy with sex hormones promotes weight gain, research has yielded conflicting results. In women, neither hormone replacement therapy after menopause (irrelevant if with estrogen/progesterone-based hormone replacement therapy or with phytoestrogens)^54,55^ nor hormonal contraception systematically lead to weight gain.^56^ On the contrary, suppression of sex hormone production in premenopausal women lowers the basal metabolic rate,^57^ suggesting even a weight-reducing effect of sex hormones. Recently, an association with increased energy intake has been found for patients treated with β-hydroxy β-methylglutaryl (HMG)-Co-A inhibitors (statins).^58^

## Influence of obesity on pharmacokinetic parameters of drugs

**Figure 2.**
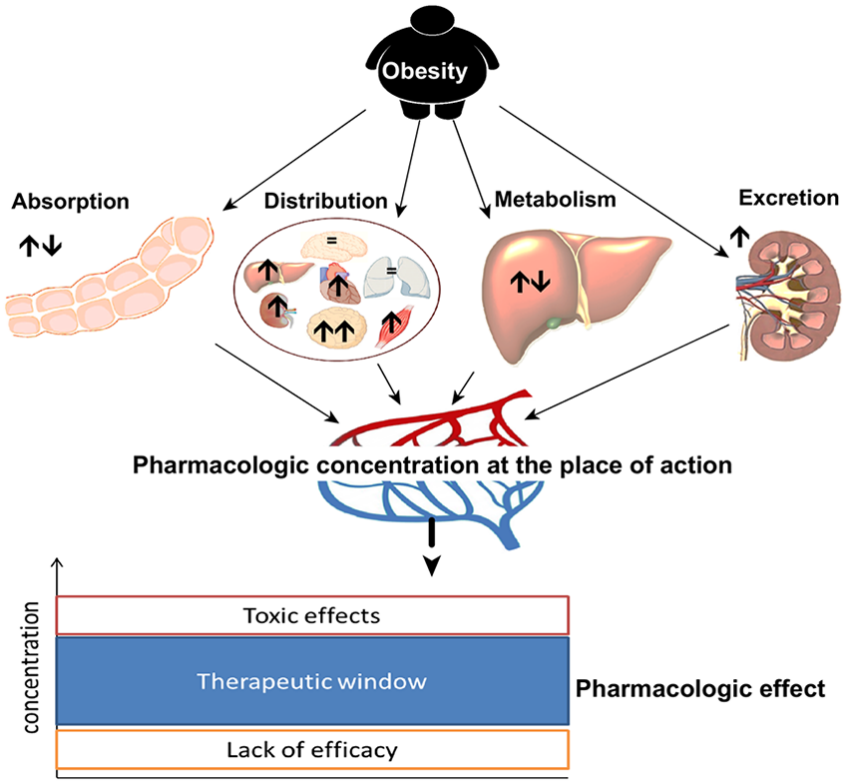
Obesity-induced changes in pharmacokinetic properties of drugs. In obese patients, pharmacokinetic drug properties are altered (arrows indicating: ↑↓ indifferent, ↑ increased, ↓ decreased capacity). Consequently, resulting drug concentration at the place of action and pharmacologic effect might be different to normal weight patients.

Body weight is determined mainly by height and the proportion of water, fat, and muscle mass. Changes in the composition of these compartments result in altered pharmacokinetic properties of drugs. The physiological body proportions of obese patients are fundamentally different from those of normal weight patients. In addition to the overly increased proportion of adipose tissue, there are also changes in regional blood flow, cardiac output is increased, body water is decreased, and relative proportion of fat-free mass per kilogram body weight is reduced.^59^ These anthropometric changes result in clinically relevant changes in pharmacokinetic and pharmacodynamic properties of drugs, as illustrated in Figure 2.^60^ Several recent reviews describe the need for an individualized therapy in obese children and adults, taking into account the described changes of body composition and physiology.^6,61,62^ Despite the high rate of obese patients worldwide, and clear indications to differences in the pharmacokinetics of many drugs, specific recommendations on weight-adjusted dosing is lacking in the product information summary of most drugs.^5^

Due to the different body composition of obese and normal weight subjects, with a disproportional high amount of adipose tissue in obese patients, it seems reasonable that drugs need to be individually dosed in obese patients, depending on their respective pharmacological and physicochemical properties. However, height, weight, or BMI of an individual do not properly reflect body composition. Bioelectric impedance analysis currently represents the simplest, fastest, most accessible, and least expensive approach to estimate lean body mass and fat mass. Alternative approaches to estimate body composition include dual energy X-ray absorptiometry, quantitative computed tomography, dilution techniques, air displacement plethysmography, three-dimensional photonic scanners, quantitative magnetic resonance, and magnetic resonance imaging and spectroscopy.^63^ Special conversion tables exist, for example, for renal insufficiency, but are currently not available for patients with obesity.^5^ To date, pharmacokinetic differences in obese patients have been poorly investigated, especially because that group of patients is often underrepresented in clinical trials.^4,5^ As yet, there is no other way but to deduce individual dosing from the pharmacokinetic properties of the respective drug.^62^ Especially for drugs with a narrow therapeutic range, therapeutic drug monitoring (TDM) should be used to assure patient safety.^5^

The pharmacokinetic properties of a drug are based on four processes: absorption, distribution, metabolism, and excretion (the ADME principle). Substantial changes in any of these processes because of the high percentage of body fat render dose adjustments necessary in obese patients.^60,62^ We summarize known obesity-associated alterations in absorption, distribution, metabolism, and excretion in the following sections.

### Absorption

Only a few studies are available in which overweight and obese individuals were studied with regard to alterations in drug absorption. Despite possible differences in intestinal blood flow and gastric emptying,^64,65^ increased body weight does not appear to be associated with clinically relevant changes in gastrointestinal absorption of drugs.^59^

An altered absorption of subcutaneous, transdermal, or intramuscular drugs is very likely in obese patients, due to an increased proportion of subcutaneous adipose tissue,^66^ and decreased local blood flow.^67^ However, the results of clinical studies are sometimes contradictory in this regard. While clinically significant differences in absorption could not be demonstrated in studies with Actrapid and low-molecular-weight heparins,^68–70^ other studies with fast-acting insulin and human choriongonadotropin showed significant absorption differences after subcutaneous injection.^71,72^ The huge amount of subcutaneous adipose tissue, and its reduced blood flow, suggest a change in bioavailability even with transdermal administration. *Inter alia*, a decreased transdermal bioavailability has been demonstrated for nicotine.^73^ In addition, application errors are possible when drugs should be injected intramuscularly; standard needles may be too short for obese people, resulting in subcutaneous injection.^74–76^

### Distribution

Distribution of drugs depends on the amount of adipose tissue, fat free mass, blood flow, and binding affinity to plasma proteins and tissue structures.^59,62^ While absolute amount of fat tissue and fat-free mass are increased in obese patients, their proportions are changed, with a decrease in the percentage of fat-free mass, and an increase in the percentage of fat mass compared with normal weight subjects.^66,77^ Cardiac output, and, thus, overall organ perfusion increase with increasing body weight,^78^ whereas relative adipose tissue blood flow decreases.^67^ There are no indications for a change in binding to plasma albumin, which is the most important drug-binding plasma protein. However, augmented binding to other plasma proteins may be possible, in particular to alpha(1)-acid glycoprotein, whose secretion is enhanced in obesity.^59^

The described changes influence the volume of distribution of drugs in obese subjects. However, a general statement about how distribution of drugs is changed in obesity is not possible.^79^ Volume of distribution of a drug depends on its pharmacological and physicochemical properties, in particular, its binding affinities.^80^ Depending on whether the drug is a lipophilic or hydrophilic molecule, different models of dose adjustment are required in the obese patient. In case of a hydrophilic drug, fat-free mass or lean body mass are crucial, whereas, for lipophilic substances, calculation of the individual therapeutic dose should be based on total body weight.^79,81^ However, this conclusion can apply only to chronic therapy. In acute short-term therapy, for example, administration of anesthetics during surgery, and dose adjustment according to total body weight, might result in overdose, even in case of drugs with high lipophilicity, which is why lean body mass is usually recommended for dose calculation during anesthesia.^82^ Likewise, lean body mass may be the most suitable reference for individual dose adjustment in overweight and obese patients in need of chemotherapy due to malignancies.^83^ However, insufficient, and often contradictory, clinical data are available, and, thus, due to concerns about potential underdosing and resulting lack of efficacy, current oncologic guidelines recommend that dose calculation is based mainly on total body weight, or body surface area calculated based on total body weight.^84,85^ Clinical trials are urgently needed to optimize drug treatment in obese oncologic patients.

### Metabolism

The liver is the most important organ for drug metabolism. Hepatic metabolism is divided in phase I and phase II reactions. Oxidation, reduction, and hydrolysis belong to phase I, and conjugation (glucuronidation, sulfation, etc.) to phase II reactions. The reactions are catalyzed by hepatic enzymes, expression of which is influenced by various factors, for example by cytokines. Many diseases, for example, inflammation, type 2 diabetes mellitus, and obesity, lead to an altered rate of hepatic metabolism.^86^ Obese patients have a reduced rate of metabolism *via* CYP3A4 isoenzymes, whereas metabolism by uridine diphosphate glucuronosyltransferases (UGT1 and UGT2 with respective subfamilies), xanthine oxidase, N-acetyltransferase, and CYP2E1 is accelerated.^87^ Moreover, activities of CYP1A2, CYP2C9, CYP2C19, and CYP2D6 might also be increased, but study results are inconsistent in this regard.^60,87^ Only limited data are available on effects of obesity on phase II reactions. Glucuronidation and sulfation of drugs appear to be induced in obese patients, which could be shown by increased clearance of paracetamol (484 *versus* 323 ml/min in men and 312 *versus* 227 ml/min in females), which is both glucuronidated and sulfated.^88^ Glucuronidation is assumed to be more strongly induced than sulfation, as shown by accelerated benzodiazepine metabolism.^89^ However, similar clearance of procainamide and salicylates in lean and obese individuals may be an indication that not all phase II reactions are altered by obesity.^90,91^

Effects of obesity on extrahepatic metabolism of drugs have been poorly studied. Besides the liver, the intestinal tract is also a very important site of drug metabolization.^92^ A connection between food intake and both intestinal CYP expression and p-glycoprotein could be found; thus, obesity-associated changes are likely to exist in intestinal drug metabolization as well.^93,94^ However, accurate data to assess the clinical relevance of these alterations are lacking. Adipose tissue itself plays only a minor role in drug metabolism. Nevertheless, a study with prednisolone revealed significant differences between healthy and obese subjects, which could not be explained by modified enzyme expression in the liver. The cause is believed to be an occurrence of 11-hydroxysteroid dehydrogenase type 1 in adipose tissue, and, thus, increased rate of extrahepatic metabolism in obesity.^95^

### Excretion

Drugs, and their metabolites, are predominantly excreted through the kidneys. Major common risk factors for chronic kidney disease are hypertension and type 2 diabetes mellitus; both are strongly associated with obesity. Thus, specific causalities for decreasing renal function in obesity are difficult to distinguish.^96^ Nevertheless, in patients with long-term obesity, renal disease is relatively common. Even if underlying mechanisms bear many similarities, obesity seems independently to promote glomerulopathies.^97^ The significant correlation of renal impairment with BMI could also be seen in a study with obese patients before bariatric surgery.^98^ Renal elimination rate is determined by glomerular filtration as well as tubular reabsorption and secretion. Various morphological and functional changes, in particular within the glomeruli, were observed after a high-fat diet and the consecutive weight gain in dogs.^99^ Initially, rapid weight gain appears to result in increased tubular sodium reabsorption and compensatory renal vasodilation. The exact mechanism for this early increase in glomerular filtration rate (GFR) is not fully understood, but, at least initially, weight gain seems to increase GFR.^100^ Whether the observed initially increased GFR is caused by weight gain is still controversially discussed. Another study in patients with morbid obesity without detectable proteinuria did not show an increased glomerular filtration rate compared with normal weight individuals.^101^ This apparent discrepancy might be explained by differences in the determination of GFR.^59^ In most cases renal function is given as the estimated GFR (eGFR). The formulas underlying the calculation are usually based on total body weight or standardized body surface area. However, these formulas are not validated for obese patients, and can be used only to a very limited extent in this population.^102,103^ This is reflected in a small study including 17 individuals with normal serum creatinine. In this comparison, GFR was not elevated in the eight obese compared with the nine normal weight subjects, when measured GFR was corrected for lean body mass. In this study, the inulin method, which allows accurate determination of GFR irrespective of body weight, was used to determine GFR.^104^

In contrast to GFR, tubular reabsorption and secretion are more difficult to measure. Nevertheless, some studies suggest possible changes in obesity. Lithium is filtered through the glomerulus and reabsorbed through tubulus cells. Therefore, increased lithium clearance in obese patients without concomitant change in GFR indicates decreased tubular reabsorption.^105^ Likewise, increased tubular secretion may lead to increased excretion of Procainamide in obesity.^90^ Confirming these findings, similar evidence for altered tubular secretion and reabsorption in obesity is available for ciprofloxacin and cimetidine.^106^

Another way of excreting drugs and drug metabolites is *via* biliary secretion. Expression of intrahepatic transport proteins, which are required for the excretion of many chemical substances, is reduced in obesity. Thus, even if only limited information is available on altered biliary secretion in obesity, an impaired biliary secretory capacity must be assumed for many xenobiotics.^107^ Study results for patients with obesity related nonalcoholic fatty liver disease are inconclusive. While liver metabolism might be decreased, at least in earlier disease stages, hepatic efflux transporter activity seems to be increased, presumably to prevent further damage by toxicants.^108^

### Specific dose recommendations in obesity

Table 2 summarizes medications for which dose recommendations in obesity are available from clinical trials. Most of the studied drugs are used only short term during anesthesia or to treat infectious diseases. Drugs used for chronic diseases often lack reliable data from pharmacokinetic studies. Dose adjustments for drugs not included in the list must be set on an individual basis, especially when drugs with a narrow therapeutic window are concerned. At least until more robust evidence becomes available, a general guide for dose adjustments can be to use total body weight as basis for dose calculation in case of highly lipophilic drugs used for short-term treatments, and lean body mass in case of chronic treatment or hydrophilic drugs.^79^ Important to note, a dose recommendation might be appropriate in individual cases, which is not covered by the respective marketing authorization and the summary of product characteristics of the drug. These cases have to be assigned to off-label use with all legal consequences.

**Table 2.** Medications with published dose recommendations in obesity.

Drug	Recommended dose adjustment
Antibiotics and antimycotics^109^	
Aminoglycosides	Ideal body weight^a^ + 0.4 (total body weight – ideal body weight), drug monitoring
Vancomycin	Total body weight, drug monitoring
Beta-lactam antibiotics	Few data, adjustment most likely according to body surface area
Daptomycin	Total body weight, a little less if appropriate
Flucytosine	Ideal body weight
Amphothericine	LBM (few data)
Tuberculostatics	Ideal body weight (few data)
Anesthetics^82,110^	
Thiopental	Induction of anesthesia: LBM
	Maintenance of anesthesia: total body weight
Propofol	Induction of anesthesia: LBM
	Maintenance of anesthesia: total body weight
Fentanyl	LBM
Remifentanil	LBM
Succinylcholine	Total body weight
Vecuronium	Ideal body weight
Rocuronium	Ideal body weight
Atracurium	Ideal body weight
Cisatracurium	Ideal body weight
Benzodiazepines	Loading dose: total body weight
	Maintenance dose: ideal body weight
Antithrombotics^111,112^	
Low molecular weight heparins	Prophylaxis: fixed-dose increased according to total body weight (few data)
	Treatment: total body weight, maximal dose in extreme obesity if necessary
Fondaparinux	Prophylaxe: fixed-dose maybe increased (few data)
	Treatment: body weight >100 kg: 10 mg daily

a Ideal body weight is the weight for which, based on a specific body height, the longest life expectancy was determined. There are a number of formulas that can be used to calculate ideal body weight, the formulas provide equivalent results.^113^

LBM, lean body mass (~ fat free mass).

## Drug treatment after weight reduction surgery

Depending on the type of surgery, surgical weight reduction interventions cause more or less dramatic anatomical changes in the gastrointestinal tract. Together with the drastic weight loss, and, thus, altered body composition, unpredictable changes in the pharmacokinetics of drugs are to be expected. A summary of the potential changes after surgery is given in Table 3.

**Table 3.** Potential physiologic changes after weight loss surgery with relevance for drug treatment.

	Physiological changes immediately after surgery^114^	Physiological changes long-term after surgery
Absorption	Alteration of gastric emptying time, gastric/gastrointestinal pH. Duodenal absorption surface reduced. Reduced intestinal first-pass metabolism (CYP3A4, CYP3A5). Decreased intestinal p-glycoprotein efflux. Decreased intestinal transit time. Dissociation of bile salt delivery.	No long-term adaption/normalization
Distribution	No immediate changes after surgery	Normalization of obesity induced alterations ^60^: ↓ fat free mass, ↓↓ fat mass, ↓ organ volume, ↓ ejection fraction, ↓ blood volume, altered protein binding, ↓ alpha-1-acid glycoprotein, ↓ plasma lipids,↓ free fatty acids
Metabolism	No immediate changes after surgery	↓ intestinal blood flow, ↑ CYP3A4, ↓ liver fat, ↓ cholestasis, ↓ periportal fibrosis, ↓ specific enzymes (uridine-diphosphate-Glucuronosyl-transferase, xanthinoxidase, N-acetyltransferase, CYP2E1), ↓ glucuronidation, sulfidation, ↓ biliary secretion/transporter activity, ↓ inflammation
Excretion	No immediate changes after surgery	Regeneration of renal insufficiency induced by obesity?

CYP, cytochrome P450.

The altered absorption conditions with often massively reduced intestinal resorption surface together with the dynamic changes of body composition and possibly altered enzyme and drug transporter activities may lead to unpredictable changes in drug efficacy, always depending on type and time after weight reduction surgery. On the other hand, disease activity of many obesity-related comorbidities improves postoperatively. There are many reviews in the literature delineating this problem, but up to now only few clinical trials are available from which clear, generally applicable clinical advice can be drawn.^114–120^ Some examples from the literature suggest that absorption and drug efficacy might be dependent on time since surgery.^121,122^ Of note, bioavailability is contrarily altered in these two examples. While bioavailability for both examined drugs normalized some months after surgery, absorption of atorvastatin was initially increased, and absorption of serotonin reuptake inhibitors was initially decreased.^121,122^ The reason for this discrepancy might be altered cytochrome P450 enzyme and transporter activities in the liver and intestine.^120^ Due to the manifold and diverse changes after weight reduction surgery, and the specific physicochemical properties of drugs, no general advice can be given on how to dose these patients correctly over time. Some smaller clinical trials have revealed some specific CYP enzyme alterations, but, unfortunately, the results are somewhat contradictory, and specific conclusions are thus difficult to draw.^123,124^ For many drugs commonly used in obese patients to treat comorbidities, such as antidepressants, antidiabetics, statins, antihypertensives, corticosteroids, contraceptives, and thyroid hormones, smaller maintenance doses are required following bariatric surgery; however, higher doses are required for other drugs.^114,118–120^ Attempts have been made to use pharmacokinetic modeling to predict plasma concentrations without groundbreaking success.^117,118^ Some clinical observations, however, reinforce the importance of the matter; for example, the observation of an increased suicide rate after bariatric surgery, which might be caused by decreased bioavailability of some antidepressants.^125^

### Altered pharmacokinetic after Roux-en-Y gastric bypass surgery

Roux-en-Y gastric bypass is one of the most popular and most effective bariatric surgery techniques. Physiological changes induced by Roux-en-Y gastric bypass influence pharmacokinetics through reduced gastric volume, decreased gastric acidity, delayed gastric emptying, reduced intestinal mucosal surface, reduced hepatic first-pass metabolism, shortened intestinal transit time, and a more distal mixing of chyme with bile acids and pancreas enzymes.^116^ Overall, drugs in liquid formulations (syrup, solution, etc.) are better absorbed than tablets, and solubility of basic drugs may be impaired while acidic drugs are absorbed more easily and faster after Roux-en-Y gastric bypass surgery. Since the intestinal transit time is also a decisive factor for the bioavailability of many drugs, and, moreover, mix with bile acids and pancreatic enzymes is delayed, dissolution of highly lipophilic drugs must be expected to be hampered, and their bioavailability to be decreased.^119^

During absorption in the intestinal mucosa, enzymatic metabolization already takes place. Moreover, there is presystemic elimination of many drugs by drug transporters such as p-glycoprotein. The capacity of these processes is severely limited postoperatively by the elimination of the proximal small intestine, and, thus, bioavailability of affected drugs may significantly increase.^118^ As mentioned above, studies examining changes in CYP activities did not present robust results and are difficult to interpret.^123,124^ There are only a few studies with sufficiently large groups of patients that have been able to demonstrate specific postoperative changes in the bioavailability of drugs after Roux-en-Y gastric bypass. The sparse literature was evaluated in several recent reviews.^114,118–120^ Available data indicate an increased bioavailability of Paracetamol and possibly Atorvastatin (shortly after surgery), a presumably unchanged bioavailability of Metformin, Morphine, Caffeine, Tolbutamide, Midazolam, Moxifloxacin, and Omeprazole, and a presumably reduced bioavailability of serotonin reuptake inhibitors, Phenytoin, Mycophenolate, Sirolimus, Tacrolimus, and Azithromycin.^114,118–120^

Overall, more research is urgently needed to optimize drug treatment after bariatric surgery, and up to now, there is no clear and simple algorithm to predict the changes in absorption kinetics of drugs after weight reduction surgery.^120^

## Conclusion

Drug treatment and body weight are linked in four ways: drugs may intentionally be used to reduce weight; drugs may unintentionally influence body weight as a side effect; increased body weight may alter the pharmacokinetic characteristics of a drug, thus requiring dose adjustment at least in massively obese patients; and efficacy of drugs in massively obese patients may be altered also due to pharmacodynamic reasons. We have provided a literature review with regards to the first three issues, with specific emphasis on discussion of the weight reduction surgery patient. Overall, a significant lack of knowledge has to be considered when a massively obese patient requires drug treatment, and many uncertainties remain. Systematic development of the clinical pharmacology of obesity is necessary to improve the safety and efficacy of drug treatment given the ever-rising numbers of this patient population.
